# Demographic Histories of ERV-K in Humans, Chimpanzees and Rhesus Monkeys

**DOI:** 10.1371/journal.pone.0001026

**Published:** 2007-10-10

**Authors:** Camila M. Romano, Fernando L. de Melo, Marco Aurelio B. Corsini, Edward C. Holmes, Paolo M. de A. Zanotto

**Affiliations:** 1 Laboratory of Molecular Evolution and Bioinformatics, Department of Microbiology, Biomedical Sciences Institute–ICBII, University of São Paulo, Brazil; 2 Mueller Laboratory, Department of Biology, Center for Infectious Disease Dynamics, The Pennsylvania State University, University Park, Pennsylvania, United States of America; 3 Fogarty International Center, National Institutes of Health, Bethesda, Maryland, United States of America; Institute of Human Virology, United States of America

## Abstract

We detected 19 complete endogenous retroviruses of the K family in the genome of rhesus monkey (*Macaca mulatta;* RhERV-K) and 12 full length elements in the genome of the common chimpanzee (*Pan troglodytes*; CERV-K). These sequences were compared with 55 human HERV-K and 20 CERV-K reported previously, producing a total data set of 106 full-length ERV-K genomes. Overall, 61% of the human elements compared to 21% of the chimpanzee and 47% of rhesus elements had estimated integration times less than 4.5 million years before present (MYBP), with an average integration times of 7.8 MYBP, 13.4 MYBP and 10.3 MYBP for HERV-K, CERV-K and RhERV-K, respectively. By excluding those ERV-K sequences generated by chromosomal duplication, we used 63 of the 106 elements to compare the population dynamics of ERV-K among species. This analysis indicated that both HERV-K and RhERV-K had similar demographic histories, including markedly smaller effective population sizes, compared to CERV-K. We propose that these differing ERV-K dynamics reflect underlying differences in the evolutionary ecology of the host species, such that host ecology and demography represent important determinants of ERV-K dynamics.

## Introduction

A considerable proportion (∼45%) of the primate genome consists of copies of mobile genetic elements [1]. These elements are divided into two classes based on their mechanism of mobilization: those involving an RNA intermediate, or those that transpose via DNA excision and reintegration into the host genome (transposons). The via-RNA elements (Class I) are represented by retrotransposons and endogenous retroviruses (ERVs). ERVs are relics of ancient viral infection events in the germ line, followed by long-term vertical transmission. They can increase in copy number by means of active replication (in *cis* or in *trans*) or by chromosomal duplication [2], and represent about 3% of all transposable elements (TE) related sequences. Proviral activity may occur over long periods of time until they become inactivated by loss of promoter functionality due to host chromosome rearrangements, insertions, deletions or point mutations. Because the LTRs (long terminal repeats) of proviruses carry transcriptional regulatory elements, such as promoters and enhancers, its likely that the insertion of a provirus, or only its LTRs, near genes or regulatory regions will be detrimental to host fitness [3]–[5].

The human ERV-K (HERV-K) family includes some of the most active retroviral elements in human genome [6], [7]. Although most of the proviral copies of ERV-K in the genome are inactive, some show evidence of past positive selection at the *env* gene [8], [9]. ERVs, as well the other retroelements, can invade the host genome due to transposition bursts [10], counteracted by host-driven excision and purging [11], [12]. This dynamical process plays an important role in the evolution of host genomes as a consequence of the rearrangement, transduction and inactivation of genes [13], [14]. In the absence of any host selection pressure to inhibit the fixation and replication, ERV copy number could increase to extreme levels [15], [16]. However, the preferential integration of LTR elements in gene-poor regions and in an antisense orientation suggests that these elements are routinely purged from gene-rich regions by purifying selection [17], [18], which is perhaps a major force restricting ERV copy number. Consequently, determining the mechanisms of transposition control, inactivation and purging are central to the understanding of proviral dynamics in the host genome [5], [12], [16], [19], [20].

To explore the evolutionary dynamics of ERVs in more detail, we determined the demographic history of ERV-K in three primates: human (*Homo sapiens*), common chimpanzee (*Pan troglodytes*) and rhesus monkey (*Macaca mulatta*). Our findings suggest that host population size and ecology plays a major role in shaping patterns of ERV-K evolution in primates.

## Results

### ERV-K Characterization and Phylogeny

Nineteen complete proviruses, designated RhERV-K, were found in the rhesus monkey (*Macaca mulatta*) draft assembly genome (Text S1). Similarly, 12 new elements in *Pan troglodytes* (CERV-K) genome were found (Text S1) and compared to 20 CERV and 55 human HERV-K previously reported, producing a total of 106 ERV-K genomes. Three RhERV-K proviruses had almost identical LTR, indicative of recent integration and therefore of possible recent activity. Conversely, RhERV-K19 had highly divergent 5′ and 3′ LTR that could not be aligned due to several insertion-deletion events (indels), indicating that the estimated integration time of about 46 MYBP (see below) may be misleading. As no RhERV-K orthologue was closely related to those in either the chimpanzee or human genomes, all RhERV-K proviruses appear to have arisen by active transposition rather than chromosomal duplication. In contrast, *Pan* and *Homo* share several ERV-K, and exhibit many closely related elements that most likely originated by chromosomal duplications and rearrangement events (*e.g.*, CERV-K32, CERV-K31, CERV-K34; CERV-K26, 27 and 28 on the Y chromosome).

**Figure 1:**
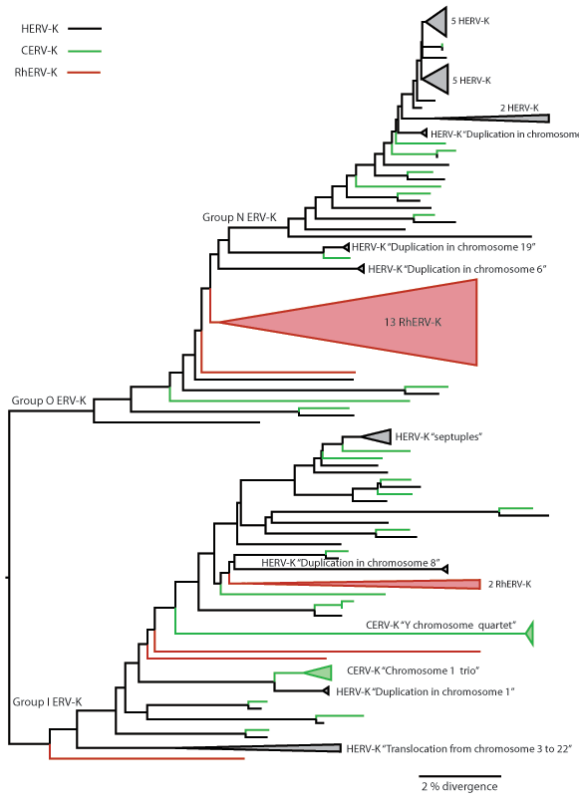
Maximum likelihood tree for 106 ERV-K genomes. ML tree for 4130 bp of shared (Partial) sequences from ERV-K genomes of human (*Homo sapiens*) (55 sequences), common chimpanzee (*Pan troglodytes*) (32 sequences) and, rhesus monkey (*Macaca mulatta*) (19 sequences). Thirteen RhERV-K (shown as a collapsed red wedge in the tree) arise from a single ancient branch in Group O, while four other deep lineages radiate independently from within Group I. No RhERV-K was observed in Group N. The HERV-K, CERV-K and RhERV-K elements are shown by black, green and red branches, respectively. Duplications of the same provirus appear in colored collapsed wedges.

A phylogenetic tree (Fig. 1) for a 4130 bp alignment from the conserved domains (the Partial data set) shared by 106 ERV-K genomes, had a topology congruent to those obtained previously for both ERV-K genomic fragments [21] and complete genomes [9]. To facilitate data presentation, tree components involving two or more adjacent lineages in the same host, were collapsed and were indicated as colored wedges in Figure 1. Human and chimpanzee appear to share a large number of ERV-K as indicated by at least 18 *Pan*-*Homo* sister taxa pairs at the tips of the tree. Interestingly, 13 RhERV-K clustered in a distinct group, radiating within Group O [9], represented by the largest wedge in Figure 1. The other six RhERV-K genomes fell in four distinct lineages within Group I. None of the six lineages of RhERV-K shared recent orthologues with *Homo* or *Pan*, and only three (RhERV-K3, RhERV-K8 and RhERV-K19) were possibly integrated into the common ancestor of all three primates. This notion was further supported by the fact that no traces of ERV-K were found in the orthologous chromosomal regions in human and chimpanzee, where we would expect to find the descendents of RhERV-K3 and the eight ERVs that predate the separation of all three lineages. Conversely, fragments of LTR and *gag* sequences were found on chromosome 9 of both human and chimpanzee at the integration site of RhERV-K19, suggesting that they the ERV-K viruses have been purged from these genomes.

### ERV-K Population Dynamics

**Figure 2:**
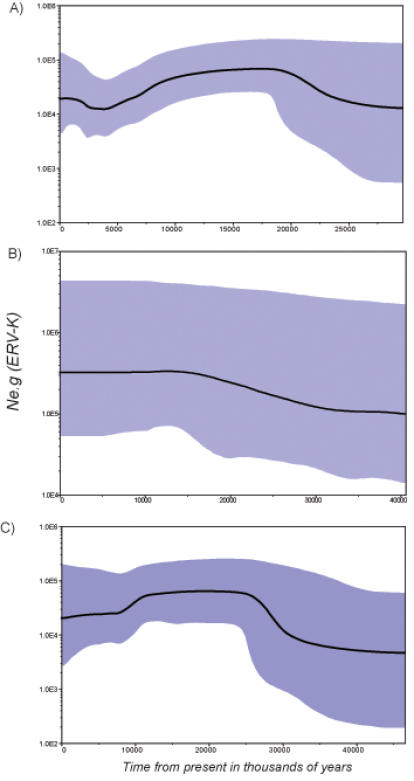
Bayesian skyline plots of three primate ERV-K. A) human (*Homo sapiens*) ERV-K (HERV-K), B) common chimpanzee (*Pan troglodytes*) ERV-K (CERV-K) and, C) rhesus monkey (*Macaca mulatta*) ERV-K (RhERV-K). Time is presented in million years from the present and effective population sizes multiplied by the generation time (*Ne*.g) are presented in a logarithmic scale on the y-axis. The bold line represents the median estimate for each species while the 95% HPDs (reflecting statistical uncertainty) are shaded. Integration times for all ERV-K were estimated using a rate of 3.3×10^−9^ substitutions per site per year (s/s/y).

Bayesian skyline plots, reflecting changes in effective population size through time, were inferred for 31 HERV-K found in *Homo sapiens* (Figure 2a), 21 CERV-K found in *Pan troglodytes* (Figure 2b) and 19 RhERV-K found in *Macaca mulatta* (Figure 2c). The high ESS values (near 1000) indicated that the sample sizes, although small, were sufficient for convergence during parameter estimation. Strikingly different plots were seen in the three species, and with a particularly complex dynamic in humans, although both *Homo* and rhesus ERV-K experienced an initial burst in ERV copy number followed by a significant reduction in the number of complete proviruses after 20 MYBP. In contrast, CERV-K experienced an apparently flat dynamic after a significantly (around ten-fold) higher growth in numbers up until 15 MYBP, and had very much larger effective population sizes than the other two species. Finally, and perhaps most notable of all, during the last 5 MY there was an increase in ERV-K numbers in the human genome, possibly caused by the radiation of the newer human elements (Group N) [9].

**Figure 3:**
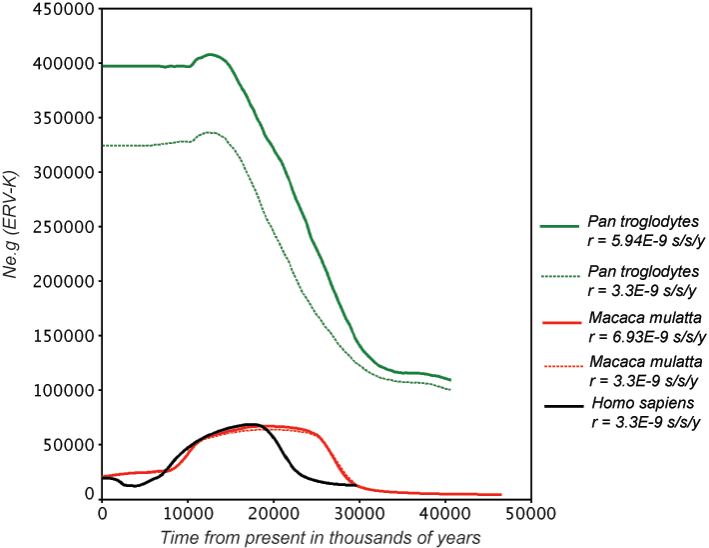
Comparative population dynamic of ERV-K. The figure shows the superimposed median values of *Ne*.g through time taken from the Bayesian skyline plots for the primate species. Time is presented in million years from the present and effective population time generation time (*Ne*.g) sizes are given in a linear scale without the 95% HPD values shown in

One possible reason for differences in the dynamics observed is heterogeneity in evolutionary rate among the primate hosts. In particular, it has been established that the rate of evolution in humans suffered a slowdown relative to that of the chimpanzee [22], [23], with an approximately two-fold reduction in evolutionary rate relative to Old World monkeys and chimpanzee [24]. Therefore, based on previous estimates on the differences among substitution rates for the species considered here [22]–[25], we repeated our analysis of population dynamics using dates of integration based on rates of 5.94×10^−9^ s/s/y for CERV-K and 6.93×10^−9^ s/s/y for RhERV-K (with human still at 3.3×10^−9^ s/s/y). The comparisons shown in Figure 3 clearly indicate that the differences in population dynamics are not changed qualitatively by host rate heterogeneity. Hence, these results indicate that the evolutionary rate of the host genome is a less important determinant of differences among ERV-Ks than host population dynamics.

## Discussion

### ERV-K in primates

Herein, we described several new complete ERV-K elements in the genomes of the common chimpanzee (*Pan troglodytes*) and rhesus monkey (*Macaca mulatta*) and compared them to those found in humans. We show, for the first time, that the demographic history of the host may be a major factor determining the dynamics of an endogenous retrovirus. Despite the draft quality of the rhesus genome assembly, we found many complete proviruses that have a marked similarity in their fluctuating demographic history to that of humans, with both these species distinct from that observed in the chimpanzee (Figure 3). In particular, we found a distinct group of 13 RhERV-K, which diverged around 12 MYBP that were absent in both humans and chimpanzees. Moreover, there was no evidence of RhERV-K amplification caused by chromosomal duplication. On the other hand, both *Homo* and *Pan* had many closely related ERV-K, some of which had several duplicated counterparts. Important differences between CERV-K and HERV-K were also evident. For example, four CERV-K where found on the Y chromosome, three of which were found within an apparently low complexity repeat region, as a consequence of DNA duplication (*i.e.*, CERV-K “Y chromosome quartet” in Figure 1). Interestingly, the human Y chromosome has the same repeat region without traces of retrovirus integration, suggesting that elements have been purged along the human lineage.

### Demography and Dynamics of ERV-K

The Bayesian skyline plots revealed fluctuating ERV-K population sizes in all three primate species, although with a relatively large sampling error (Figures 2 and 3). Although HERV-K and RhERV-K had similarly complex skyline plots, it is striking that the latter exhibited a signal of rapid population growth up until 25 MYBP, coinciding with both fossil and molecular data for the radiation of the *Cercopithecidae*. Conversely, the signal for the initial burst for HERV-K and CERV-K occurred at approximately 17–18 MYBP, followed by a reduction of the number copy of the elements, first in *Homo* and then in *Pan*. This growth signature, common to all three primates, may reflect some of the shared history of ERV-K colonization of Catarrhines from the Oligocene (30 MYBP) to Miocene (20 MYBP).

The rate of retrovirus-driven transposition and excision is evidently insufficient to explain their permanence and integrity. Since, in finite populations, size fluctuations have a drastic impact on genome architecture, ERV-K numbers in time must ultimately depend on host population dynamics [26]. Nevertheless, the mechanisms of purging [5], reduction of transposition efficiency by APOBEC [20], excision [11] and stabilization under weak selection [16], or the balance between host migration rates and ERV-K transposition rates [12], as well as synergistic epistasis among integrated ERV-K [27], may have played a role in preventing the continued growth of the three ERV populations towards the present from 10 to 20 MYBP. The loss of cladogenetic signal from older ERV-K lineages could therefore be a consequence of a strong host-driven purging that is more evident in the *Homo* and rhesus lineages. This agrees with our finding that 61% percent of the human elements compared to 21% of the chimpanzee and 47% of rhesus had estimated integration times less than 4.5 MYBP.

Since all partial sequences we dismissed were likely generated by incomplete purging events it is evident that our approach has underestimated the loss of ERV proviruses. Nevertheless, by investigating complete genomes were able to estimate integration times, which is only possible when both LTRs are present. The Bayesian skyline plot for HERV-K showed a conspicuous population bottleneck in the last 17 MY, comprising a significant reduction in complete proviral numbers up until 4MYBP, after which a cladogenetic burst within ERVs from Group N [9] took place. This population bottleneck could indicate a recent loss of ancient signal in the hominids, since the difference in the skyline signatures predates the split of *Homo* and *Pan*. Possibly, bottlenecks since the Plio-Pleistocene may have played an important role, facilitating both the loss of unfixed alleles and the fixation of deleterious ones by genetic drift [28], and which could help explain the observed complex dynamics of HERV-K. Intriguingly, the time frame for a “re-colonization” of the hominids by Group N HERV-K at around 1.5 MYBP coincides with the emergence of human-specific life history traits [23], such as increased generation time.

Unlike the single extant species of the genus *Homo*, the genus *Macaca* is represented by a large number of species (19) despite being a relative young clade [29], [30]. *Macaca mulatta* originated from a *fascicularis*-like ancestor around 2.5 MYBP and became widely distributed within a relatively short period, from western India to the eastern coast of China. The strong decrease in RhERV-K population size (Figure 3) coincided with the emergence of the genus *Macaca* around 10 MYBP, which is one of the most specious groups among *Cercopithecidae* [29]. The impact of the intense cladogenesis in *Cercopithecidae* on RhERV-K dynamics remains to be addressed. Nevertheless, the elevated dispersal of both *Homo* and *Macaca* compared to *Pan* may be an important factor that could explain the similarities in the demographic histories of HERV-K and RhERV-K.

Unlike HERV-K and RhERV-K, the chimpanzee ERV-K demographic signal was characterized by a far larger effective population size. Assuming that host dynamics impacts on ERV-K numbers, the recent flat curve of *Pan* skyline after 6 MYBP agrees with the lack of evidence for severe bottlenecks in the *Pan* lineage and a 3.2 times larger effective ancestral population size [31]. The latter could have facilitated the maintenance of a higher number of integrated elements observed in the chimpanzee genome, because of a weaker effect of genetic drift, although the wide HPD values caution against over-interpretation.

## Methods

### ERV Screening, Phylogenetic Inference and Sequence Analysis

We screened the genomes of *Pan troglodytes* (build 2 v.1) and the *Macaca mulatta* draft assembly (v.1) by BLAT search [32] using complete ERV-K genomes as a query. This analysis revealed 116 complete retroviral genome sequences, 78 of which were previously reported and are deposited in GenBank as DQ112093-DQ112156. These sequences were then aligned with both MUSCLE [33] and BlastAlign [34]. To minimize systematic errors caused by insertion/deletion events (indels), for which there is no adequate model of evolution, we also constructed a 4130 bp data set using gene coding regions only (designated as the ‘Partial’ data set from now on). Maximum likelihood (ML) trees of these data were then inferred by PAUP v.4.0b [35], using the TVM+Γ evolutionary model as determined by MODELTEST 3.7 [36]. Tree topologies were evaluated from an initial neighbor joining tree (NJ), using a heuristic search approach that implemented successively branch-swapping methods: (*i*) tree bisection-reconnection (TBR) branch-swapping, (*ii*) subtree pruning-regrafting (SPR) and, (*iii*) nearest-neighbor interchange (NNI). The integration time (T) of each provirus was estimated using the relation *T* = *d*/2*r*, where *d* is the genetic distance between 5′ and 3′ LTR and *r* is the rate of nucleotide substitution per site. Errors in *T* where assumed to be the transformed values of the standard errors for *d* estimations. Because rates of substitution for ERVs can range from 1.5–5×10^−9^ substitutions per site per year (s/s/y), [2], [37] we used an average rate of 3.3×10^−9^. Finally, pairwise distances among ERVs were calculated using Tamura-Nei model available in MEGA2 [38].

### Population Dynamics

For this analysis we constructed a smaller 2530 bp region from the Partial dataset that contained those nucleotide sites shared by all proviruses. Proviruses that were both sister taxa (*i.e.* adjacent in the phylogenetic tree) and had similar flanking regions up to10 kb away from the insertion locus, were excluded from the demographic analyses as they most likely to have arisen by chromosomal duplication. Following this screening, 19 RhERV-K, 21 CERV-K and 31 HERV-K sequences were available for analysis. Rates of nucleotide substitution per site, the time to the Most Recent Common Ancestor TMRCA and the demographic history of each ERV-K group (*Homo*, *P. troglodytes* and *M. mulatta*) were estimated using a Bayesian Markov Chain Monte Carlo (MCMC) method available in the BEAST package [39]. For this analysis, dates of integration based on LTR distances were used as “sampling dates” since, once integrated, ERV-K proviruses would behave as if they were “frozen” in the genome and so evolve at rates equivalent to those of host DNA. Such LTR-based “sampling dating” is justified since the differences in the rates of evolution of exogenous retroviruses are six orders of magnitude higher than those of their endogenous (“frozen”) counterparts. Because LTR comparisons indicate that ERV-K have been integrating into primate DNA for at least 40 million years, the assumption that all ERV-K were sampled today would entail a far greater systematic error. To infer population dynamics of the different primate ERV-K we fitted sequence data to the demographic models available in the Bayesian coalescent method in BEAST. In particular we used the Bayesian skyline plot to depict changes in effective population size through time (N_e_.*g*, where N_e_ is the effective population size and *g* the generation time). For this analysis we used the HKY+Γ model of nucleotide substitution under the assumption of a relaxed (uncorrelated exponential) molecular clock. The HKY+Γ was consistently the best-supported model in MODELTEST when the data from each species were analyzed separately. In all cases chain lengths of 40–50 million were sufficient to obtain Effective Sample Sizes (ESS) greater than 100.

## Supporting Information

Text S1 GenBank information
(0.03 MB DOC)
