# Supplementary material for: Demographic Histories of ERV-K in Humans, Chimpanzees and Rhesus Monkeys
Source: PLoS One. 2007 Oct 10;2(10):e1026. doi: 10.1371/journal.pone.0001026 (PMC2001186; doi:10.1371/journal.pone.0001026)
Supplement: Text S1 — GenBank information (0.03 MB DOC) [file pone.0001026.s001.doc]

**Supporting Information S1**

The nineteen *Macaca mulatta* ERV-K (RhERV-K) genomes found in this work are in GenBank under accession numbers: NW_001108926, NW_001108771, NW_001108971, NW_001096629, NW_001098162, NW_001102952, NW_001104443, NW_001106513, NW_001112540, NW_001116480, NW_001118153, NW_001118154, NW_001121136, NW_001122906, NW_001218142, NW_001116520, NW_001114224, NW_001121000, NW_001124240.

The twelve new *Pan troglodytes* ERV-K (CERV-K) genomes can be found by searching the following contigs: NW_001229127, NW_001240645, NW_001229126, NW_001223147, NW_001252915, NW_00125919, NW_00125925, NW_001240316, NW_001235338, NW_001234057, NW_001234039, NW_001234091.
